# Exploring the effect of environmental sensitivity on emotional fluctuations among adolescents during the COVID-19 pandemic: a three-wave longitudinal study

**DOI:** 10.3389/fpsyg.2024.1443054

**Published:** 2024-07-22

**Authors:** Mirella Dragone, Alessandra Colella, Concetta Esposito, Dario Bacchini

**Affiliations:** ^1^Faculty of Law, Giustino Fortunato University, Benevento, Italy; ^2^Department of Humanities, University of Naples “Federico II”, Naples, Italy

**Keywords:** Environmental Sensitivity, adolescence, COVID-19 pandemic, emotions, longitudinal study

## Abstract

**Introduction:**

The COVID-19 pandemic significantly impacted the emotional well-being of adolescents worldwide. Some studies suggested that individuals with high Environmental Sensitivity may have been more likely to experience poor psychological adjustment during the pandemic than those with lower sensitivity. However, there is still limited research on how emotional responses varied across different stages of the pandemic and whether Environmental Sensitivity increased adolescents’ vulnerability to the psychological impact of prolonged pandemic restrictions.

**Methods:**

To address this gap, this study used a three-year longitudinal design (2020–2022) with a sample of 453 adolescents. They completed an online survey measuring their positive and negative emotions throughout the pandemic period, with Environmental Sensitivity considered a time-invariant covariate.

**Results:**

The results revealed that all participants, regardless of their level of Environmental Sensitivity, experienced a decrease in positive emotionality between the first and second years. However, this trend reversed, showing an increase between the second and third years. Regarding negative emotions, highly sensitive adolescents experienced a significant linear increase over time. In contrast, low-and medium-sensitive adolescents exhibited a quadratic trend, with a notable increase in negative emotions between the first and second years, followed by a slight decrease between the second and third years.

**Discussion:**

These findings provide further evidence of the negative impact of the pandemic on adolescents’ emotional well-being. They also support the notion that Environmental Sensitivity is associated with individuals’ ability to respond and adjust to adverse life events, with significant implications for future research and practice.

## 1 Introduction

The outbreak of the novel coronavirus disease (COVID-19) raised a global impact worldwide, leading to a mental health crisis for all populations (Arora et al., 2022). The home-confinement and social distancing measures implemented to prevent the spread of the virus have had a significant impact on people’s daily routines, becoming a potentially stressful life event, especially for adolescents (Rogers et al., 2021). Adolescents experienced high and stable rates of COVID-19 life disruption over the course of the pandemic, albeit with some differences across cultural contexts (Rothenberg et al., 2024). Their regular activities, such as attending school, socializing with friends, and participating in recreational activities, have been suddenly disrupted. This disruption occurred during a crucial period of development, marked by rapid physical, emotional, and cognitive changes. Experiences during this period lay the foundation for adult identity and behavior (Imran et al., 2020; Grusec and Davidov, 2021) and disruptions that occurred during the pandemic might have potentially interfered with the acquisition of crucial skills like independence, forming peer relationships, and future planning (Sarkadi et al., 2021; Carey et al., 2023). Research has shown that adolescents have exhibited signs of irritability, likely influenced by increased parental involvement limiting privacy and alone time (Hasking et al., 2021). Additionally, high levels of stress have been reported, likely as a result of concerns about safety and education during the pandemic, particularly with the sudden shift to online learning (Hasking et al., 2021). Physical distancing has also led to increased loneliness, potentially due to concerns about maintaining social connections as before (Ellis et al., 2020). These daily-life challenges, together with the greater propensity of adolescents to experience more intense emotions, with higher frequency and volatility (Bailen et al., 2019) may have put them at risk for poor physical and mental health during the pandemic, leading to anxiety disorders, depression, and suicidal behavior (Meherali et al., 2021; Saulle et al., 2022; Elharake et al., 2023; Panchal et al., 2023).

However, it is worth noting that the effects of the pandemic on individuals were not homogeneous, as individuals’ reactions to it varied. Several studies have shown that individual differences played a role in how people responded to the COVID-19 pandemic (Zettler et al., 2022). Therefore, the roles of interindividual differences in people’s cognitive, emotional, and behavioral responses to the pandemic have been and remain subject to intensive exploration. For instance, some studies have shown that individuals with high levels of neuroticism were more worried about COVID-19 and experienced greater stress during the pandemic (Aschwanden et al., 2021; Liu et al., 2021). Additionally, Modersitzki et al. (2021) found that facets of extraversion, neuroticism, and openness were among the strongest and most important predictors of negative psychological outcomes, even after controlling for gender and age.

This study aims to investigate how Environmental Sensitivity (ES), which refers to differences among individuals in how they perceive and process environmental stimuli (Pluess, 2015), related to adolescents’ emotional experiences during the pandemic. Although research has explored the effects of ES on individuals who have experienced childhood adversities (Karam et al., 2019; May et al., 2024), there remains a gap in understanding its impact on adolescents in stressful and emergency situations. To date, very few studies (Bordarie et al., 2022; Güneş and Bulut, 2022; Lionetti et al., 2023) have explored the role of ES in capturing individual differences in adapting to the challenges associated with the COVID-19 pandemic, with a limited focus on adolescents (Burgard et al., 2022; Iimura, 2022). This study aims to delve into the emotional experience of highly sensitive individuals in times of crisis, with the goal of gaining insights into how they navigate environmental stressors.

### 1.1 COVID-19 pandemic and adolescents’ emotional well-being

Numerous studies have examined the impact of the pandemic on the psychological well-being of adolescents, and all have concurred on the rise of emotional difficulties among children and adolescents following the pandemic outbreak and spread. Zhou et al. (2020) conducted one of the earliest studies on the effects of the pandemic on adolescent mental health, with Chinese participants aged 12 to 18. The study reported a high incidence of depressive symptoms (43%), anxiety (37%), and a combination of both (31%). Similarly, Wang et al. (2020) found that roughly 30% of participants showed symptoms related to anxiety, while 17% displayed symptoms related to depression. These findings are consistent with conclusions drawn from other studies in China (Duan et al., 2020; Ma et al., 2023) as well as from various regions around the world (see for review, Elharake et al., 2023; Panchal et al., 2023). Additionally, several studies have found that girls and older adolescents tended to struggle more with emotional issues, reporting a higher risk of experiencing negative emotions such as anger and sadness (Schwartz et al., 2021; Strasser et al., 2023), and problems such as depression and stress, compared to boys and younger adolescents, respectively (Magson et al., 2021; Schwartz et al., 2021; Kauhanen et al., 2023; Morales-Vives et al., 2024).

Despite the increasing number of longitudinal studies, there is still a lack of understanding of how emotional issues have developed during and after the pandemic emergency, especially among adolescents, and whether adolescents have recovered from the challenges posed by the pandemic. Some of these longitudinal studies have compared pre-pandemic well-being with well-being during the pandemic. In a systematic review conducted by Kauhanen et al. (2023) of 21 studies published until October 2020, including more than 96,000 subjects up to 24 years of age before and during the COVID-19 pandemic, the authors found a longitudinal deterioration in symptoms for different mental health outcomes in adolescents and young people. Research conducted by Shoshani and Kor (2022) examined the impact of the COVID-19 pandemic on children and adolescents’ mental health and subjective well-being in Israel. The study analyzed data from the beginning of 2019 until the end of 2020, revealing concerning findings. Specifically, the results showed higher levels of general distress and an increase in internalizing disorders such as anxiety and depression, as well as panic symptoms, compared to the pre-pandemic period. Additionally, participants reported a decrease in positive emotions, lower life satisfaction, and reduced peer support compared to the time before the virus spread and quarantine measures were implemented. Two reviews from German cohort studies in 2020 described an average doubling of anxiety (21, 26%) and depression (25, 29%) during the pandemic compared to the pre-pandemic period (Vogel et al., 2021; Schlack et al., 2023).

Other studies have investigated the trajectories of adolescent well-being at different points during the pandemic. Asscheman et al. (2021) conducted a daily diary study examining mood fluctuations during the first four weeks of the pandemic (April–June 2020) and found relatively stable mood levels in early adolescents in the Netherlands. This finding partially aligns with a study by Orgilés et al. (2022), which assessed children and adolescents in Italy, Spain, and Portugal during an eight-week period at the onset of the pandemic. The study revealed that Portuguese children, who adhered to a general duty of home confinement, adapted best to the situation with no significant changes over time, while Italian participants were more psychologically affected by home confinement. Surprisingly, Spanish children exhibited a significant decrease in anxiety symptoms by the last assessment. As the authors argued, these variations could be explained by the differing levels of restrictive measures implemented in each country. Minihan et al. (2024) conducted a study at three-month intervals between May 2020 (T1) and April 2021 (T3), involving a sample of over 3,000 participants aged from 11 to 100 years. The study found that younger age was linked to more frequent use of maladaptive emotion regulation strategies. This, in turn, was associated with more negative affect at T3, controlling for the negative affect at T1. The study by Foster et al. (2023) revealed that COVID-19-related stress during the lockdown period in 2020 predicted subsequent symptoms of anxiety and depression in adolescents during the summer of 2021. Further studies generally confirm this trend (e.g., Polack et al., 2021; Houghton et al., 2022).

To our knowledge, there is only one study that has tracked the course of psychological adjustment in children and adolescents throughout the pandemic until nearly the end of the crisis (Ravens-Sieberer et al., 2023). In their large five-wave longitudinal study conducted from January 2020 to September–October 2022 in Germany with children and adolescents aged 7 to 17 years (known as the COSPY study), Ravens-Sieberer et al. (2023) found that after two years of pandemic-related deterioration in child and adolescent mental health, there was finally an improvement in 2022. More in detail, there were slight improvements in autumn 2021, but progress leveled out in winter 2021/22. As the authors stated, these improvements in mental health could be attributed to an ongoing adaptation process during the past pandemic years, such as increasing resilience, the resumption of normal social, physical, and entertainment activities due to fewer restrictions, the availability of vaccinations making disease courses less severe, and the fact that most children coped well with the COVID-19 infection itself.

Overall, the literature emphasizes the need for more research into how adolescents’ emotional responses changed during the different stages of the COVID-19 pandemic. It also suggests exploring the potential differential impact on various groups based on individual differences, which has not been thoroughly addressed so far. Taking individual differences into account may provide valuable insights into effectively supporting their coping mechanisms against the emotional challenges they encounter.

### 1.2 Environmental sensitivity and psychological adjustment

Based on empirical evidence and theoretical reasoning, individuals may differ in their response to environmental influences, with a minority of the population, around 25–30% of individuals (Lionetti et al., 2018; Pluess et al., 2018) being highly sensitive to the quality of their environment, for better and for worse (Greven et al., 2019; Pluess et al., 2023). Such individual differences in sensitivity to environmental influences could be captured by the temperamental trait of Environmental Sensitivity, an umbrella term encompassing theories that explain individual differences in the ability to register and process environmental stimuli (Pluess, 2015; Pluess et al., 2023). This concept is rooted in three primary theoretical frameworks: Sensory Processing Sensitivity Theory (Aron and Aron, 1997), Differential Susceptibility Theory (Belsky, 1997; Belsky and Pluess, 2009), and Biological Sensitivity to Context Theory (Boyce and Ellis, 2005). These frameworks have been integrated into a comprehensive Environmental Sensitivity meta-framework from the perspective of developmental psychology (Pluess, 2015).

Individuals with heightened Environmental Sensitivity possess a more sensitive central nervous system, characterized by specific brain region activation (Acevedo et al., 2014) that facilitates deeper processing of sensory input (Aron et al., 2012; Greven et al., 2019). Consequently, they tend to exhibit heightened awareness of environmental information and engage in more complex and thorough processing compared to individuals with lower sensitivity levels. This affects the way they plan, think, and learn and makes them more susceptible to the quality of their surroundings, which in turn affects their overall development.

This heightened sensitivity has been conceptualized within several theoretical frameworks, including the diathesis-stress model, which posits that highly sensitive individuals are more vulnerable to adverse experiences (Belsky and Pluess, 2009); the differential susceptibility theory, which suggests that sensitivity operates as a susceptibility factor, making individuals not only more likely to be adversely affected by negative environments but also to benefit more from supportive ones (Belsky and Pluess, 2009; Ellis and Boyce, 2011; Belsky, 2013); and vantage sensitivity, which highlights the tendency of highly sensitive individuals to benefit from particularly positive experiences (Pluess and Belsky, 2013; de Villiers et al., 2018).

Investigations into Environmental Sensitivity within the general population have linked it to various mental health and behavioral consequences, such as reduced subjective happiness (e.g., Sobocko and Zelenski, 2015), heightened stress levels (e.g., Bakker and Moulding, 2012), and less effective coping mechanisms for stress (e.g., Brindle et al., 2015). Conversely, research evidencing the bright side of Environmental Sensitivity, where individuals benefit disproportionately from positive experiences, is still scarce (Greven et al., 2019).

Overall, there is limited research on psychological adjustment to stressful situations within the Environmental Sensitivity meta-framework, particularly among adolescents, as most studies focus on childhood (Greven et al., 2019). Among the few existing studies involving adolescents, Karam et al. (2019) analyzed the impact of childhood adversities and war exposure on Post-Traumatic Stress Disorder (PTSD) symptoms in young Syrian refugees aged 7–17 years. The findings revealed that the effects of the Syrian war on PTSD were most pronounced in children with high sensitivity and low childhood adversities and least pronounced in children with low sensitivity and low childhood adversities. These results indicate that Environmental Sensitivity plays a crucial role in moderating PTSD symptoms, particularly in the absence of other chronic adverse developmental conditions, such as unsupportive family environments. Additionally, Rubaltelli et al. (2018) found that after viewing terrorism-related images, highly sensitive individuals (university students) were more willing to compromise their privacy compared to those in neutral experimental conditions. Conversely, among less sensitive individuals, no significant difference was observed. These findings support the notion that individuals with higher Environmental Sensitivity perceive a higher level of threat when exposed to images depicting terrorist attacks due to their deeper processing of sensory information and heightened emotional and behavioral reactivity triggered by such exposure (Aron et al., 2012).

Furthermore, in their retrospective study, Di Paola et al. (2023) examined in a sample of Italian young adults aged from 18 to 30 years the interplay between individual differences in Environmental Sensitivity and resilient contexts defined as social and family support in buffering the impact of childhood experiences of neglect on adulthood relational well-being. The authors found that highly sensitive individuals who experienced childhood emotional neglect were more affected by the benefit of a resilient context, increasing their level of relational well-being as compared to low-sensitive adults.

Drawing from these findings and consistent with previous empirical evidence highlighting that highly sensitive individuals show a stronger emotional/physiological reactivity and a feeling of overstimulation in response to excessive demands from the environmental context (Aron and Aron, 1997; Aron et al., 2012), it is possible that the COVID-19 pandemic, as a large-scale stressor, could have resulted in adaptation difficulties and psychological distress, especially for highly sensitive individuals. Additionally, with the relaxation of pandemic restrictions and the changing circumstances, there is an opportunity to explore how highly sensitive adolescents have benefited from the more positive experiences following the peak of the pandemic.

### 1.3 Environmental sensitivity and COVID-19 pandemic

So far, only a small number of studies have explored the role of Environmental Sensitivity in the context of the COVID-19 pandemic (Bordarie et al., 2022; Güneş and Bulut, 2022; Lionetti et al., 2023; Van Landeghem and Jakobson, 2024), with a limited focus on adolescents (Burgard et al., 2022; Iimura, 2022). Overall, such studies provided evidence that the dispositional trait of Environmental Sensitivity might capture individual differences in response to the environment during the COVID-19 pandemic. Van Landeghem and Jakobson (2024) investigated the impact of several personality traits and childhood emotional abuse on symptoms of anxiety and depression among university students during the pandemic. They found that all these variables were associated with feeling more negatively impacted by the COVID-19 pandemic in daily life. Notably, they demonstrated for the first time that sensory processing sensitivity was the most significant personality trait in explaining individual differences in perceived COVID-19 impacts and that females felt these impacts more strongly compared to males. This finding aligns with and supports previous research by Güneş and Bulut (2022) and Bordarie et al. (2022), who found a significant effect of sensory processing sensitivity on the perceived impact of COVID-19 on health anxiety and general anxiety/depression disorders and quality of life, respectively.

In addition, in a longitudinal study with a sample of school-aged children, Lionetti et al. (2023) explored the interplay between parenting quality and Environmental Sensitivity on children’s behavioral adjustment during and immediately after, the COVID-19 lockdown restrictions. They found that highly sensitive children, who were more at risk of internalizing problems before the COVID-19 outbreak, showed lower levels of internalizing behaviors compared to pre-COVID-19 in the presence of a highly supportive parent–child relationship.

To the best of our knowledge, only two studies have explored the role of Environmental Sensitivity in adolescence. Burgard et al. (2022) used a cross-sectional design to examine a sample of children and adolescents aged 9 to 18 years during the first lockdown in the Netherlands. They found that sensory processing sensitivity was associated both directly and indirectly—through the perceived impact of COVID-19—with higher internalizing problems, such as depression and anxiety. Similarly, in a cross-sectional study with a sample of older adolescents and young adults from Japan, Iimura (2022) observed that those with high sensory processing sensitivity experienced elevated levels of stress related to COVID-19 in older adolescents and young adults.

Taken together, these findings suggest that variations in Environmental Sensitivity could have served as a vulnerability factor during the COVID-19 pandemic, particularly influencing negative emotionality. However, there is no research investigating how Environmental Sensitivity influenced adolescents’ emotional well-being when conditions improved. This emphasizes the need to study the impact of Environmental Sensitivity during adolescence across different stages of the pandemic.

### 1.4 The present study

The COVID-19 pandemic has had a significant impact on Italy, with notably high rates of positive cases and mortality in 2020 (Villani et al., 2020). In response to the crisis, the Italian government implemented two national lockdowns (March–May 2020 and October–November 2020) and a three-tiered system of restrictions based on transmission rates and impact on older age groups and healthcare. These measures led to an unusual and prolonged period of stress, especially impacting the lives of children and adolescents, which in turn affected their quality of life and overall well-being (Nocentini et al., 2021; Barbieri et al., 2023; Bonvino et al., 2023).

This context is particularly concerning given that adolescence is a period characterized by volatile emotional states and heightened sensitivity to social and emotional changes, largely due to pubertal maturation (Steinberg, 2005). In general, it is widely recognized that adolescents undergo greater fluctuations in both positive and negative emotions compared to children and adults, with emotional reactivity peaking during mid-adolescence (Larson et al., 2002). Additionally, there is a general trend across adolescence of increased negative emotions and decreased positive emotions (Bailen et al., 2019).

However, the impact of the COVID-19 pandemic on these developmental patterns has been little explored, with existing studies being predominantly cross-sectional. For instance, Strasser et al. (2023) conducted a study with a large sample of students from kindergarten to 12th grade in North Chile, collecting data in 2020. They found that high school and female students exhibited more negative emotions compared to middle school and male students, respectively. In another study, Calma-Birling and Zelazo (2022) performed two network analysis studies on two different samples of high school and college students in the U.S., each at a different time during the pandemic. The aim of the study was to explore risk and resilience factors associated with students’ negative and positive emotions during the first year of the COVID-19 pandemic. They found that concern about conflict with parents was the pandemic-related concern most strongly connected to negative affect, while self-compassion was most strongly connected to positive affect. Descriptively, high levels of negative affect were reported during 2020, with a slight and significant decrease in 2021. Conversely, positive affect mean values showed a significant slight increase over time.

Despite these findings, there is still limited research on how emotional responses varied across different stages of the pandemic and whether Environmental Sensitivity made adolescents more vulnerable to the psychological impact of prolonged pandemic restrictions. This study aims to address these gaps by examining the impact of the COVID-19 pandemic on fluctuations of positive and negative emotions in a sample of Italian adolescents. Additionally, it will use the Environmental Sensitivity theoretical framework to predict how adolescents responded during the pandemic. Furthermore, the role of gender and age was explored.

Based on the literature and research goals, the following hypotheses are posited:

*H1*: Adolescents are expected to show a complex pattern of positive emotions throughout the different phases of the COVID-19 pandemic. Initially, positive emotions may decrease during the start of pandemic-related restrictions and lockdowns (T1-T2). Then, as these restrictions ease and the pandemic conditions improve, positive emotions may stabilize or slightly increase (T3).

*H2*: Similarly, negative emotions among adolescents are anticipated to follow a multifaceted pattern over time. Negative emotions might intensify during the initial period of strict lockdown measures (T1), peak during subsequent waves of pandemic-related restrictions (T2), and then gradually decrease as restrictions ease and societal conditions improve (T3).

*H3*: From a developmental perspective, older adolescents, representing late adolescence, may demonstrate more pronounced fluctuations in both positive and negative emotions compared to their younger counterparts, reflecting their increased need for independence and social interaction.

*H4*: Gender differences are expected, with female adolescents likely to report higher levels of negative emotions and lower levels of positive emotions compared to male adolescents across various stages of the pandemic.

*H5*: Highly sensitive adolescents are anticipated to experience a greater decrease in positive emotions and a larger increase in negative emotions during periods of strict lockdown measures (T1 and T2). Given the scarcity of studies, no hypotheses are proposed as pandemic-related restrictions gradually ease (T3).

No hypotheses were formulated regarding the effects of age and sex across different sensitivity levels.

## 2 Materials and methods

### 2.1 Study design and sample

This study employed a three-wave longitudinal design spanning from 2020 to 2022, with a one-year interval between each wave. To avoid any confusion, it is important to note that the survey waves of the study do not coincide with the waves of COVID-19 infections, as will be described below.

The research involved 453 Italian high school students (156 males and 297 females, with an average age at T1 of 15.91, *SD* = 1.32) who were requested to complete an online survey. The participants were drawn from seven high schools in the metropolitan area of Naples, situated in southern Italy.

Data collection took place in March–April 2020 (Time 1; T1), during the initial COVID-19 wave and national lockdown. Between T1 and T2, there was an intermediary period over the summer when restrictions were eased, but in October 2020, the restrictions were made stricter again because of a new peak of infections. The second wave of data collection occurred in March–April 2021 (Time 2; T2), when schools reopened following the introduction of the vaccine, and students alternated between in-person and online classes while adhering to mask mandates and other COVID-19 preventive measures. The third wave of data collection was in March–April 2022 (Time 3; T3), when restrictions were significantly eased, allowing for a return to normal day-to-day activities.

Schools were recruited from a university-school network. The school directors were initially contacted via email and invited to take part in the study. Parental consent for student participation was then requested through traditional school-family communication channels. Additionally, students provided their online assent before beginning the questionnaire. The data collection at T1 was conducted during virtual classes, with trained researchers providing instructions and guidance remotely. For the data collection at T2 and T3, when students were physically present, the survey was administered in person by the researchers. The study was approved by the Psychological Ethics Committee of the University Department (Protocol code n. 21/2020).

### 2.2 Missing data and attrition analysis

Over the study period, 453 participants were initially assessed at T1. At T2, 428 participants were re-evaluated, reflecting a 6% attrition rate. At T3, only 116 participants remained in the study, resulting in a total attrition rate of 74%. This attrition rate is near the commonly reported range in longitudinal studies, typically between 30 and 70% (Gustavson et al., 2012). In the current study, the decrease in participation was primarily attributed to participant drop-out or loss of contact with the school following the peak of the pandemic period. Despite this attrition, Little’s test for Missing Completely at Random (MCAR) was not significant, χ^2^(8) = 6.576, *p* = 0.58, thus indicating that the missing data does not follow any systematic pattern and can be considered random. Additionally, t-tests conducted as part of the missing data analysis revealed no significant differences between those who dropped out and those who remained in the study across key demographics (age, gender) and other study variables (all *p*s > 0.05). Given the limitations of t-tests, which may not detect differences in distribution shapes, density plots comparing cumulative distributions of demographic and study variables between dropout and remaining participants are available at https://osf.io/vfy9g/?view_only=546914fa64bf4c72b4900875e252bba1 for further support.

### 2.3 Measures

#### 2.3.1 Positive and negative affect

The Positive and Negative Affect Schedule (PANAS; Watson et al., 1988) was used to measure emotional states at all three time points in this study. The PANAS assesses two dimensions of affect:

Positive Affect (PA), which captures high-arousal states of pleasure and engagement, such as excitement and happiness. A high score on PA reflects high energy, more concentration, and pleasurable engagement, while a low score is associated with sadness and lethargy (Watson et al., 1988); Negative Affect (NA), which reflects high-arousal states of aversive emotionality. It represents a general dimension of subjective stress and displeasure, encompassing various aversive mood states such as anger, contempt, disgust, guilt, fear, or nervousness. A low NA score indicates a state of calm and tranquility (Watson et al., 1988).

We used 20 items, each rated on a response scale from 1 (Not at all) to 5 (Very much). Positive emotional states are measured by 10 items (e.g., joy, interest, and enthusiasm), with Cronbach’s alpha ranging from 0.77 to 0.84 across T1 to T3 (T1 = 0.77, 95% C.I. [0.74, 0.80]; T2 = 0.77, 95% C.I. [0.75, 0.80]; T3 = 0.84, 95% C.I. [0.83, 0.87]). Negative emotional states are measured by 10 items (e.g., distress, sadness, and fear), with Cronbach’s alpha ranging from 0.77 to 0.81 across T1 to T3 (T1 = 0.77, 95% C.I. [0.74, 0.80]; T2 = 0.76, 95% C.I. [0.73, 0.79]; T3 = 0.81, 95% C.I. [0.78, 0.83]).

#### 2.3.2 Environmental sensitivity

The study used the Highly Sensitive Child Scale (HSC; Pluess et al., 2018) to measure Environmental Sensitivity at Time 1 (T1). The HSC consists of 12 items, each rated on a response scale from 1 (Not at all true) to 5 (Completely true). A sample item from the scale is “I notice when small things have changed in my environment.” The scale demonstrated acceptable reliability, with a Cronbach’s alpha of 0.74 (95% C.I. [0.71, 0.77]).

Following the evidence suggesting that sensitivity is normally distributed, and people generally fall into three distinct sensitivity groups from low (bottom 30% of HSC scores) to medium (40%) to high (top 30% of HSC scores) sensitivity (Lionetti et al., 2018), a grouping variable reflecting the three sensitivity groups (low, medium, and high) was created and used in the analyses.

### 2.4 Analytic plan

The analytic plan involved several steps to identify the best-fitting trajectory of emotional fluctuations and assess potential differences based on ES. Latent Growth Curve Models (Duncan et al., 2006; Curran et al., 2010) were used. Initially, three unconditional models were tested: Random-Intercept Only Model (No Growth Model), which assumes that individuals’ emotional fluctuations do not change over time and only includes a random intercept, representing individual variability around a single mean trajectory; Linear Growth Model, which assumes that emotional fluctuations change linearly over time. It includes a random intercept and slope parameters to capture linear changes in emotional fluctuations across time; Quadratic Growth Model, which assumes that emotional fluctuations change nonlinearly over time, following a quadratic trajectory. In addition to the random intercept and slope parameters from the linear model, this model includes a quadratic term to capture the curvature of emotional fluctuations across time. The Bayesian Information Criterion (BIC) was then used to compare the trajectory models. The BIC values were computed to identify the model with the lowest BIC as the preferred model, indicating better fit relative to other models considered.

After determining the best-fitting trajectory, the analysis proceeded to assess possible differences based on ES groups in emotional fluctuations. This was achieved through multi-group analysis, which involves comparing the parameters of the trajectory model across different groups defined by levels of ES. More specifically, four models were compared using BIC values and differences in −2 Log Likelihood to assess potential differences across groups (Grimm et al., 2016): In Model 1 (M1 – Fully Constrained Model), all parameters, including means, variances, and residuals, were constrained to be equal across groups. This means that no differences in the growth trajectories of emotional fluctuations were allowed between groups based on ES. In Model 2 (M2 – Partially Constrained Model), variances and residuals were constrained to be equal across groups, while means were allowed to vary. This allowed for differences in the average levels of emotional fluctuations between groups but not in the variability or residual errors. In Model 3 (M3 – Partially Constrained Model), variances were constrained to be equal across groups, but residuals were allowed to vary. This model allowed for differences in the residual errors of emotional fluctuations between groups while keeping variances equal. In Model 4 (M4 – Unconstrained Model), no constraints were imposed across groups. Means, variances, and residuals were all allowed to vary freely, enabling the detection of any potential differences in the growth trajectories, variability, and residual errors of emotional fluctuations between groups.

In all models, the effects of age and sex assigned at birth (1 = male, 2 = female) were examined by including both variables as predictors of the intercepts and slopes. Missing data were handled using Full Information Maximum Likelihood (FIML) estimation.

## 3 Results

### 3.1 Descriptive statistics and bivariate correlations

Table 1 presents the descriptive statistics and bivariate correlations for the study variables. The correlations were calculated using the pairwise method to maximize available information across varying sample sizes over time. The results showed that Environmental Sensitivity was positively correlated with negative emotionality at all three time points and with sex, with girls reporting higher scores compared to boys. Positive emotionality at all time points was significantly negatively correlated with negative emotionality at corresponding time points. Additionally, sex was significantly correlated with both positive and negative emotionality, indicating potential gender differences in emotional experiences. Specifically, girls reported lower levels of positive emotionality at T2 and T3, along with higher levels of negative emotionality across all assessment points, compared to boys. Age had a modest yet statistically significant correlation with both negative emotionality at T1 and positive emotionality at T3. This indicates that older adolescents experienced slightly elevated negative emotions during the COVID-19 outbreak and diminished positive emotionality when restrictions were lifted.

**Table 1:** Descriptive statistics and bivariate correlations among the study’s variables.

	Mean	*SD*	1	2	3	4	5	6	7	8	9
Sex (female)			--								
Age	15.91	1.32	0.08	--							
Environmental sensitivity	3.39	1.32	0.26	0.05	--						
Positive emotionality – T1	3.06	0.74	−0.09	−0.06	−0.02	--					
Positive emotionality – T2	2.80	0.65	−0.13	−0.06	−0.09	0.53	--				
Positive emotionality – T3	3.07	0.77	−0.27	−0.20	−0.15	0.38	0.49	--			
Negative emotionality – T1	2.86	0.89	0.27	0.10	0.36	−0.25^*^	−0.25	−0.04	--		
Negative emotionality – T2	3.20	0.83	0.37	0.08	0.36	−0.22	−0.32	−0.32	0.60	--	
Negative emotionality – T3	3.17	0.88	0.40	0.15	0.35	−0.18	−0.24	−0.49	0.47	0.63	--

The significance of each correlation coefficient (𝑟) can be determined by comparing its absolute value to the critical value corresponding to the degrees of freedom (𝑑𝑓 = 𝑁−2) at the 0.05 significance level. The critical values for the sample sizes at different time points are as follows: Measures at T1: 𝑁 = 453, 𝑑𝑓 = 451, Critical value ≈ 0.092; Measures at T1 and T2: 𝑁 = 428, 𝑑𝑓 = 426, Critical value ≈ 0.098; Measures at T1, T2, and T3: 𝑁 = 116, 𝑑𝑓 = 114, Critical value ≈ 0.196.

### 3.2 Latent growth curve models

The latent growth curve analysis revealed non-linear growth patterns for both positive and negative emotionality during the COVID-19 pandemic (Table 2). Specifically, the quadratic growth model provided the best fit for both emotional states, as indicated by the lowest BIC compared to the no-growth and linear models. At T1, adolescents reported an average score of 3.06 out of 5 for positive emotionality. This score decreased by 0.57 units for each subsequent time unit, but the decrease became less steep at a rate of 0.30 over time, suggesting a potential shift toward an increase at T3 (Table 3).

**Table 2:** Comparison of latent growth curve models.

	Positive emotionality			Negative emotionality		
	No growth	Linear	Quadratic*	No growth	Linear	Quadratic*
χ^2^ (df)	106.655 (6)	84.335 (3)	3.301 (2)	108.630 (6)	28.932 (3)	2.128 (2)
CFI	0.53	0.62	0.99	0.58	0.89	0.99
RMSEA	0.17	0.22	0.03	0.18	0.12	0.01
BIC	2658.072	2654.837	**2580.164**	3158.231	3097.617	**3077.174**

*Variance and covariances of the quadratic slope were constrained to zero due to model identification requirements. Preferred models, indicating the best fit to the data, are highlighted in bold font.

**Table 3:** Growth parameters from the latent growth curve analysis.

	Positive emotionality			Negative emotionality		
	*B*	*SE*	*p*	*B*	*SE*	*p*
Intercept	3.06	0.03	<0.001	2.82	0.04	<0.001
Linear slope	−0.57	0.06	<0.001	0.55	0.07	<0.001
Quadratic slope	0.30	0.03	<0.001	−0.20	0.04	<0.001

For negative emotionality, adolescents initially reported an average score of 2.82 out of 5. This score increased by 0.55 points linearly over time, with the rate of increase slowing down by 0.20 units, indicating an initial rise that decelerated at T3 (Table 3).

When adding age and sex as covariates, the results indicated that sex was the sole predictor of the positive emotionality intercept, with girls displaying lower scores on positive emotionality compared to boys (*B* = −0.13, *p* = 0.033). Furthermore, age and sex had a significant effect on the negative emotionality intercept, with female and older adolescents exhibiting higher scores on negative emotionality (*Bs* = 0.46 and 0.06, *p* < 0.001 and *p =* 0.037, respectively).

### 3.3 Multiple-group latent growth models

The comparison among the multiple-group latent growth curve analysis revealed that the fully constrained model (M1) had the best fit for positive emotionality, thus suggesting that the growth trajectories of positive emotionality do not differ across low, medium, and high Environmental Sensitivity groups (Table 4). For all groups, positive emotionality starts at an average value of 3.06, decreases initially by 0.52 units per time unit, and then the rate of decline slows down and turns into an increase, as indicated by the quadratic term of 0.26 (Table 5).

**Table 4:** Fit statistics for the multiple-group linear growth models.

	Model M1 (fully constrained)	Model M2 (means allowed to vary across groups)	Model M3 (means and variances allowed to vary across groups)	Model M4 (Means, variances, and residuals allowed to vary across groups)
Positive emotionality				
Parameters	**7**	13	19	21
-2LL	**1953.678**	1943.836	1935.654	1933.904
BIC	**1996.489**	2023.342	2051.856	2062.338
Δ parameters		6	6	2
Δ-2LL		9.84	8.18	1.75
*p-value*		0.13	0.23	0.42
Negative emotionality				
Parameters	7	**13**	19	21
-2LL	2285.92	**2220.918**	2217.222	2.215.60
BIC	2328.732	**2300.425**	2333.425	2344.038
Δ parameters		**6**	6	2
Δ-2LL		**65.00**	3.70	1.62
*p-value*		**<0.001**	0.72	0.45

Preferred models, indicating the best fit to the data, are highlighted in bold font.

**Table 5:** Growth parameters from the multiple-group latent growth curve analysis – positive emotionality.

	Positive emotionality		
	*B*	*SE*	*p*
Intercept	3.06	0.03	<0.001
Linear slope	−0.52	0.06	<0.001
Quadratic slope	0.26	0.04	<0.001

**Figure 1:**
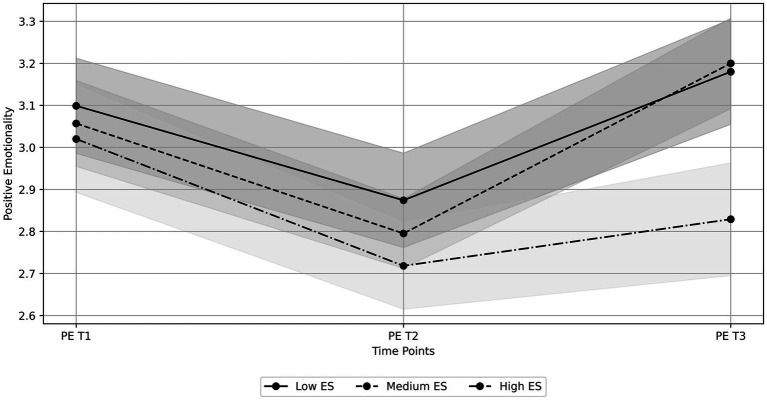
Observed trajectories of positive emotionality at three time points (PE T1, PE T2, and PE T3) for groups with low, medium, and high Environmental Sensitivity (ES). The lines represent the estimated mean scores for each group at each time point, while the shaded areas denote the 95% confidence intervals. For the low sensitivity group, mean scores with confidence intervals are: T1 = 3.10, C.I. [2.99, 3.21], T2 = 2.87, C.I. [2.76, 2.99], and T3 = 3.18, C.I. [3.05, 3.31]. For the medium sensitivity group, mean scores with confidence intervals are: T1 = 3.06, C.I. [2.95, 3.16], T2 = 2.80, C.I. [2.71, 2.88], and T3 = 3.20, C.I. [3.09, 3.31]. For the high sensitivity group, mean scores with confidence intervals are: T1 = 3.02, C.I. [2.89, 3.15], T2 = 2.72, C.I. [2.61, 2.82], and T3 = 2.83, C.I. [2.69, 2.96].

**Figure 2:**
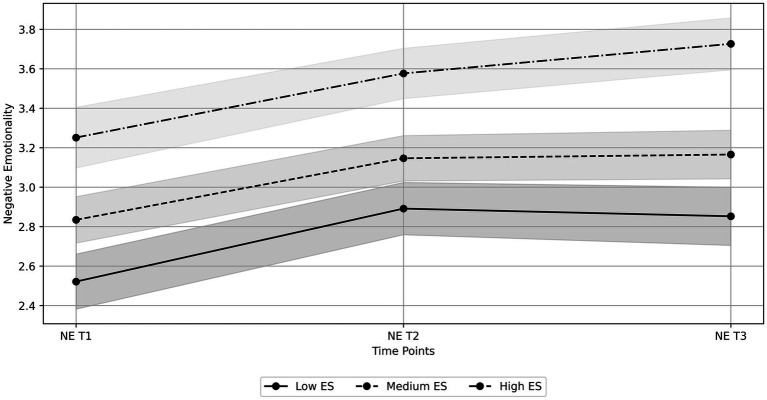
Observed trajectories of negative emotionality at three time points (NE T1, NE T2, and NE T3) for groups with low, medium, and high Environmental Sensitivity (ES). The lines represent the estimated mean scores for each group at each time point, while the shaded areas denote the 95% confidence intervals. For the low sensitivity group, mean scores with confidence intervals are: T1 = 2.52, C.I. [2.38, 2.66], T2 = 2.89, C.I. [2.76, 3.02], and T3 = 2.85, C.I. [2.70, 3.00]. For the medium sensitivity group, mean scores with confidence intervals are: T1 = 2.83, C.I. [2.72, 2.95], T2 = 3.15, C.I. [3.03, 3.26], and T3 = 3.17, C.I. [3.04, 3.29]. For the high sensitivity group, mean scores with confidence intervals are: T1 = 3.25, C.I. [3.10, 3.40], T2 = 3.58, C.I. [3.45, 3.70], and T3 = 3.73, C.I. [3.59, 3.86].

Conversely, the model with variances and residuals constrained to be equal across groups and means allowed to vary across groups (M2) had the best fit for negative emotionality, thus indicating distinct means’ patterns across groups (Table 4). For the Low Sensitivity group, negative emotionality starts at 2.52 and initially rises by 0.57 units per time unit. The rate of increase slows down over time (quadratic term: −0.20), resulting in a slight decline after the initial increase. In the Medium Sensitivity group, negative emotionality starts at 2.83 and initially rises by 0.46 units per time unit. The rate of increase also slows down over time (quadratic term: −0.15), leading to a slight decline after the initial increase. Finally, in the High Sensitivity group, negative emotionality starts at 3.25 and rises steadily by 0.43 units per time unit. The quadratic term is not significant (−0.10), indicating a primarily linear increase without a significant deceleration or decline (Table 6). The observed trajectories for positive and negative emotionality, represented with estimated mean scores and their respective confidence intervals across groups, are depicted in Figures 1, 2, respectively.

**Table 6:** Growth parameters from the multiple-group latent growth curve analysis – negative emotionality.

	Negative emotionality		
	*B*	*SE*	*p*
Low sensitivity group			
Intercept	2.52	0.07	<0.001
Linear slope	0.57	0.13	<0.001
Quadratic slope	−0.20	0.08	<0.05
Medium sensitivity group			
Intercept	2.83	0.06	<0.001
Linear slope	0.46	0.11	<0.001
Quadratic slope	−0.15	0.07	<0.05
High sensitivity group			
Intercept	3.25	0.07	<0.001
Linear slope	0.43	0.13	<0.001
Quadratic slope	−0.10	0.08	0.19

When examining the role of age and sex, the results indicated that neither sex nor age had significant effects on the initial level, linear growth, or quadratic growth of positive emotionality across low, medium, and high sensitivity groups (all *p*s > 0.05). However, when analyzing negative emotionality, distinct effects across groups emerged. In the Low Sensitivity group, sex had a significant positive effect on the intercept (*B* = 0.41, *p* = 0.004), indicating that girls reported higher initial levels of negative emotionality. Age had a significant negative effect on the linear slope (*B* = −0.21, *p* = 0.036), suggesting that older adolescents experienced a smaller increase in negative emotionality. For the quadratic slope, both age and sex had a significant negative effect (*B*s = −0.35 and 0.16, *p* = 0.025 and *p* = 0.013, respectively), signifying that the rise in negative emotionality decelerates more for females and older adolescents. In the Medium Sensitivity group, sex had a significant positive effect on the intercept (*B* = 0.41, *p* < 0.001), indicating higher initial levels of negative emotionality for girls, with no other significant effects observed. No other significant effect was found. In the High Sensitivity group, neither sex nor age had significant effects on the intercept, linear slope, and quadratic slope (all *p*s > 0.05).

## 4 Discussion

Framed within the Environmental Sensitivity meta-framework (Pluess, 2015; Pluess et al., 2023), the current study contributed to the literature on the long-lasting effects of the COVID-19 pandemic. It investigated the emotional well-being of a group of high school students at three different time points over a period of two years, corresponding to various stages of governmental interventions in response to the pandemic, based on infection and mortality rates. Also, in line with previous research (e.g., Burgard et al., 2022; Iimura, 2022) highlighting the role of Environmental Sensitivity as a significant marker of differential susceptibility to the psychological impact of the pandemic, we tested whether the changes in emotionality across different stages of the pandemic varied depending on the adolescents’ level of Environmental Sensitivity. The study also took into account the effects of the sex assigned at birth and age.

Consistent with our hypotheses (H1 and H2) and the notion that adolescence is marked by increased emotional volatility and heightened sensitivity to social and emotional changes (Steinberg, 2005; Bailen et al., 2019), we observed a significant quadratic trend in both positive and negative emotionality over time in the entire sample. Specifically, adolescents showed a significant decrease in their positive emotionality between the first and second waves of the pandemic, followed by an increase between the second and third waves; a trend toward a decrease in negative emotionality was observed. These findings align with those reported by Minihan et al. (2024), which showed an increase in negative affect over time, and with Foster et al. (2023), who revealed that COVID-19-related stress during the first pandemic lockdown predicted subsequent anxiety and depression symptoms in adolescents during the summer of 2021. Also, they are consistent with those of Ravens-Sieberer et al. (2023), who monitored changes in the well-being of children and adolescents from January 2020 to September–October 2022. Like Ravens-Sieberer et al. (2023), both positive and negative emotionality returned to more typical levels by the last assessment point after initially worsening.

These fluctuations in emotional reactions over the course of the pandemic likely reflect changing external conditions, such as infection and mortality rates, and government policies. The first wave of the pandemic was characterized by the novelty of the experience, hope for a rapid solution, and efforts to activate coping strategies. In contrast, subsequent waves lacked these characteristics, triggering a greater sense of hopelessness, helplessness, and an inability to see an end to the pandemic. The sharp initial decrease in positive emotionality and increase in negative emotionality at the beginning of the pandemic can be attributed to COVID-19-related worries, such as fear of contagion, online learning difficulties, limited in-person peer interactions, lack of emotional connection with friends, conflicts with parents, and pre-existing emotional vulnerabilities (Rogers et al., 2021). The tendency to recover during the later stages of the pandemic may be explained as a result of an adaptation process to pandemic challenges and the increasingly decreasing infection and death rates.

Furthermore, as some authors argue (Li et al., 2021), this return to normal levels of both positive and negative emotionality could be attributed to a “psychic numbing” process (Slovic, 2007). Over time, people become less sensitive to the pandemic’s deleterious effects, making them less likely to experience negative emotions and more likely to experience positive ones. Additionally, maintaining strong negative emotions over an extended period is too costly to psychological well-being. Unpleasant feelings generally motivate actions or thoughts to avoid those feelings (Epstein, 1994), such as re-evaluating the risk of infection as less dreadful than initially thought.

As concerns the role of sex assigned at birth and age, the results partially supported both H3 and H4, as well as findings from previous research (e.g., Schwartz et al., 2021; Morales-Vives et al., 2024). Regarding H3, which posited that older adolescents would demonstrate more marked fluctuations in both positive and negative emotions compared to younger counterparts, our findings revealed a significant effect of age on negative emotionality. Specifically, older adolescents exhibited higher scores on initial levels of negative emotionality, indicating a greater propensity for experiencing negative emotions compared to younger adolescents. This aligns with the developmental perspective that late adolescence, representing older age groups, may indeed be characterized by heightened emotional volatility, reflecting increased needs for independence and social interaction (Larson et al., 2002). However, the rate of change over time did not result associated with age, meaning that all adolescents, independent of their age, displayed the same fluctuations in negative emotionality across the pandemic years. Furthermore, our results also support H4, which predicted gender differences in emotional responses to the pandemic. Consistent with this hypothesis, the study revealed that sex was a significant predictor of both positive and negative emotionality. Specifically, girls exhibited lower scores on initial levels of positive emotionality and higher scores on negative emotionality compared to boys. This aligns with previous research findings by Strasser et al. (2023), who observed that female students reported more negative emotions compared to male students.

In line with research that acknowledges the dispositional trait of Environmental Sensitivity as an influential factor in emotional well-being during the pandemic (e.g., Van Landeghem and Jakobson, 2024), and partially consistent with our fifth hypothesis (H5), we found variations in emotional fluctuations among adolescents based on their level of Environmental Sensitivity. Specifically, the results of the multiple-group latent growth curve analyses indicated that all individuals, regardless of their Environmental Sensitivity, demonstrated a decrease in positive emotionality between the first and second waves of the pandemic, followed by an increase between the second and third waves. Therefore, contrary to our expectations, we found no significant differences in the change of positive emotionality over time across the groups. Conversely, we observed a noteworthy linear increase in negative emotionality over time for highly sensitive adolescents. In contrast, low—and medium-sensitive adolescents exhibited a quadratic trend, with a significant increase between the first and second waves followed by a tendency to decrease between the second and third waves.

Such discrepancies in changing positive and negative emotionality depending on adolescents’ levels of Environmental Sensitivity may reflect the notion that positive and negative feelings are believed to operate independently from each other, with changes in positive feelings informing us little about changes in negative ones, and *vice-versa* (Watson and Tellegen, 1985). These results also support the idea that individual differences in how adolescents perceive and process environmental stimuli can influence their ability to respond and adjust to adverse life events (Pluess, 2015), such as the pandemic. They provide further evidence that highly sensitive individuals are more likely to experience adverse effects during the COVID-19 pandemic (Burgard et al., 2022; Iimura, 2022). Additionally, our findings contribute to the existing literature by demonstrating that the easing of restrictions might have provided a significant positive change, improving positive emotionality even in adolescents with high Environmental Sensitivity, despite their continued higher levels of negative emotionality. The Differential Susceptibility theory (Belsky, 1997; Belsky and Pluess, 2009) helps explain why adolescents high in Environmental Sensitivity showed a continuous increase in negative emotionality but did not differ in positive emotionality improvements compared to their low-sensitivity peers. These adolescents are more reactive to environmental stressors, leading to a linear increase in negative emotionality during prolonged negative conditions. They also tend to engage in more ruminative thinking, especially when the quality of the environment is less than optimal (Lionetti et al., 2022). Due to their heightened ability to perceive and process environmental stimuli (Pluess, 2015; Pluess et al., 2023), highly sensitive adolescents may have been more attuned to the contextual changes brought about by the pandemic compared to their less sensitive peers. This heightened awareness of the negative aspects of the pandemic situation could have contributed to a sustained increase in negative emotions. However, when the environment becomes more positive, even those highly sensitive can experience an uplift in positive emotions, indicating that positive changes can have a broad and relatively uniform impact on well-being across different levels of sensitivity. This highlights Environmental Sensitivity as a susceptibility factor rather than merely a vulnerability, in line with findings from a recent study by Iimura (2021). Exploring the relationships between weekly life events and weekly socioemotional well-being in a sample of adolescents, the authors found that, in some weeks, adolescents with high sensitivity were more likely to benefit from both negative and positive events than those with low sensitivity.

The examination of the effects of age and sex across the sensitivity groups revealed significant effects of both covariates on the initial level of negative emotionality, the rate of increase over time, and the change in the rate of increase in the low sensitivity group. Specifically, girls reported higher initial levels of negative emotionality. Older adolescents showed a smaller increase over time, and both female and older adolescents showed a deceleration in the rise of negative emotionality.

These findings highlight the complexity of the relationship between individual characteristics and emotional experiences, emphasizing the need for further research to understand the mechanisms underlying these associations.

While much of the research within the Environmental Sensitivity meta-framework (Pluess, 2015; Pluess et al., 2023) has primarily emphasized its “dark” side, portraying it mainly as a vulnerability factor, our findings underscore the importance of recognizing the potential benefits associated with this dispositional trait. As evidenced by several studies, sensitive individuals are indeed more susceptible to environmental influences, both positive and negative. This heightened susceptibility makes them more vulnerable to adverse experiences (e.g., Karam et al., 2019), yet concurrently more likely to derive advantages from a nurturing and supportive environment (e.g., Di Paola et al., 2023), as well as intervention programs (e.g., Dragone et al., 2022). Consequently, further investigation is needed into how Environmental Sensitivity interacts with positive changes occurring in the environment. Future studies might delve into the dynamics of how different stages of recovery or improvement in conditions impact sensitive individuals, thus providing a comprehensive understanding of the susceptibility spectrum.

Furthermore, drawing inspiration from these findings, prevention efforts should pay particular attention to highly sensitive adolescents to maximize the benefits of programs aimed at promoting their mental health during and after stressful events like the pandemic. The disruptions caused by COVID-19 put adolescents at risk for increased negative emotions. Therefore, professionals should take a multifaceted approach to environmental and contextual factors to optimize intervention effects and prevent or mitigate the pandemic’s impact on the psychological well-being of young people.

## 5 Strengths, limitations, and future directions

The current study has several strengths and limitations that need to be acknowledged. The longitudinal design is a notable strength as it helps mitigate biases associated with generalizing the initial impact of the pandemic over time and provides insights into adolescents’ emotional states across different stages of the pandemic over two years. However, it is important to note that the absence of pre-pandemic measures of emotionality does not guarantee that the findings are specific to the challenges following the pandemic outbreak rather than reflecting developmental aspects or the natural progression of time. Additionally, the study’s strong theoretical grounding in individual differences within the Environmental Sensitivity meta-framework offers valuable insights into the individuals’ diverse reactions to the pandemic.

Also, our study, like most longitudinal studies carried out in the context of the pandemic, might have suffered from a significant attrition effect, which specifically concerned the third assessment point (i.e., the transition from T2 to T3, after two years following the outbreak of the pandemic). Future research in emergency situations might benefit from employing strategies to mitigate attrition, such as enhanced participant engagement or more flexible data collection methods.

Among the limitations, it should be acknowledged that all measurements in the study relied solely on adolescent self-reporting, which may be subject to social desirability bias. Utilizing a multi-informant approach, such as incorporating parents’ reports for temperamental constructs, along with a multi-method approach (such as combining quantitative and qualitative measures), could provide a more comprehensive understanding of emotional experiences in the context of a pandemic. Another limitation is the generalizability of the results, as the study included a sample from a limited geographic area in Southern Italy, potentially leading to selection bias. It is important to recognize that the impact of the COVID-19 pandemic may have varied among different subgroups within the Italian population and in other countries with varying infection and death rates and restriction rules.

Finally, the study does not consider the role of other contextual-level variables that may buffer the impact of the COVID-19 pandemic on adolescents’ emotional well-being. In line with the Differential Susceptibility Model (Belsky and Pluess, 2009), according to which Environmental Sensitivity should not be considered a vulnerability factor *tout court* but rather a potential advantage or disadvantage depending on the rearing environment (Lionetti et al., 2023), examining the moderating role of supportive environments like nurturing parenting, could provide valuable insights into the association between Environmental Sensitivity and emotional well-being.

## Data availability statement

The datasets presented in this study can be found in online repositories. The names of the repository/repositories and accession number(s) can be found at: https://osf.io/vfy9g/?view_only=546914fa64bf4c72b4900875e252bba1.

## Ethics statement

The studies involving humans were approved by Ethical Committee of Psychological Research, Department of Humanities, University of Naples “Federico II.” The studies were conducted in accordance with the local legislation and institutional requirements. Written informed consent for participation in this study was provided by the participants’ legal guardians/next of kin.

## Author contributions

MD: Conceptualization, Data curation, Formal analysis, Investigation, Methodology, Writing – original draft, Writing – review & editing. AC: Data curation, Investigation, Methodology, Writing – original draft. CE: Conceptualization, Data curation, Formal analysis, Investigation, Methodology, Writing – original draft, Writing – review & editing. DB: Conceptualization, Investigation, Methodology, Project administration, Supervision, Writing – review & editing.
